# Potential for social involvement modulates activity within the mirror and the mentalizing systems

**DOI:** 10.1038/s41598-017-14476-9

**Published:** 2017-11-02

**Authors:** Chiara Begliomini, Andrea Cavallo, Valeria Manera, Cristina Becchio, Roberto Stramare, Diego Miotto, Umberto Castiello

**Affiliations:** 1 Department of General Psychology, University of Padova, Padova, Italy; 2 Cognitive Neuroscience Center, University of Padova, Padova, Italy; 3 Department of Psychology, University of Turin, Turin, Italy; 4 CoBTeK, Université de la Côte d’Azur, Côte d’Azur, France; 5 STARS, Institut national de recherche en informatique et en automatique, Côte d’Azur, France; 6 Cognition, Motion and Neuroscience Unit - Fondazione Istituto Italiano di Tecnologia, Genova, Italy; 7 Department of Medicine, University of Padova, Padova, Italy; 8 Centro Linceo Interdisciplinare, Accademia dei Lincei, Rome, Italy

## Abstract

Processing biological motion is fundamental for everyday life activities, such as social interaction, motor learning and nonverbal communication. The ability to detect the nature of a motor pattern has been investigated by means of point-light displays (PLD), sets of moving light points reproducing human kinematics, easily recognizable as meaningful once in motion. Although PLD are rudimentary, the human brain can decipher their content including social intentions. Neuroimaging studies suggest that inferring the social meaning conveyed by PLD could rely on both the Mirror Neuron System (MNS) and the Mentalizing System (MS), but their specific role to this endeavor remains uncertain. We describe a functional magnetic resonance imaging experiment in which participants had to judge whether visually presented PLD and videoclips of human-like walkers (HL) were facing towards or away from them. Results show that coding for stimulus direction specifically engages the MNS when considering PLD moving away from the observer, while the nature of the stimulus reveals a dissociation between MNS -mainly involved in coding for PLD- and MS, recruited by HL moving away. These results suggest that the contribution of the two systems can be modulated by the nature of the observed stimulus and its potential for social involvement.

## Introduction

During social interactions, people engage in active social perception processes, such as attending to others’ body motion (BM), to evaluate the social situation. For instance, approach is associated with more positive evaluations than avoidance behavior, and a handshake preceding social interaction enhances the positive impact of approach and diminishes the negative impact of avoidance behavior on the evaluation of social interactions^1^. As another example from everyday experience, we know that when someone has their back turned their social attitude is much reduced compared with when they are facing us. This social cue is not just unique to humans. The behaviour of animals is sensitive to whether another individual is present or not and also to whether that individual is or is not facing them, or looking at them^2–4^.

Despite its crucial role in guiding everyday social interactions, little is known about the social nature of BM signals and how our brain codes these signals. Here, we investigated how our brain reacts while observing a model approaching and moving away from the viewer. Furthermore we were interested in exploring whether the awareness of dealing with a biological rather than a non-biological agent can significantly change our attitude towards a possible social interaction. To this aim we compared Point-Light Displays (PLD)^5^ with Human-Like stimuli (HL). PLD allow to represent kinematics of human movements by depicting activity via a few moving light points and to extract multiple aspects of social information including interactions with other people^6–10^. For instance, Manera and colleagues^11^ have investigated how well people can distinguish between different social intentions by observing a reach-to-grasp movement reproduced through PLD. The results show that PLD essential kinematic patterns were indeed sufficient to discriminate between individual and social intentions driving the action.

Another study examined the link between perceived body orientation and motor simulation processes^12^, using bistable PLD to assess the presence of any bias in the perception of stimulus direction (approaching/moving away). Results showed that the stimulus tended to be perceived as facing the viewer when participants were performing the same action (walking) as the PLD, suggesting that motor simulation mechanisms may be more salient when observed actions are directed towards the observer. Altogether, these findings emphasize the modulating effect of a potential social interaction assigned to PLD.

Evidence coming from neuroimaging suggests that regions of the Mirror Neurons System (MNS)^13^ are activated in response to point-light human motion^14,15^. The MNS includes the ventral PreMotor Cortex (vPMC), the posterior Inferior Frontal Gyrus (pIFG), the Superior Temporal Gyrus (STG) and the rostral Inferior Parietal Lobule (rIPL)^14,16–18^ and is characterized by a “mirror” mechanism in the sense that the same set of neurons is activated both when a person performs an action and when he/she observes another person performing the same action^19^. The study of Saygin^14^ measured brain activity while participants were observing PLD, matched scrambled biological motion and stationary point-light images, and showed that the observation of point-light biological motion activates regions of the frontal cortex, with particular reference to the premotor cortex, while the observation of scrambled biological motion does not. These findings suggest that the motor system of the observer may be recruited to “fill in” these simplified displays, in a manner similar to the way mirror neurons are activated in order to assist in action understanding. In a similar vein, Ulloa and Pineda^15^ examined whether the recruitment of the MNS, as reflected in *mu* rhythm suppression, mediates recognition of point-light biological motion. Changes in *mu* power were recorded while participants viewed PLD, matched scrambled versions of PLD, and visual white-noise (baseline). The results revealed that PLD produced *mu* suppression relative to baseline, while scrambled versions of these animations did not, corroborating the hypothesis that the MNS is involved in inferring human actions by recovering object information from sparse input.

Although an involvement of the MNS in the processing of PLD has been demonstrated, whether the MNS is sensitive to the potential for social involvement carried by such stimuli remains an open question. In this respect, some have advanced the hypothesis that the MNS works in concert with the Mentalizing System (MS), including regions along the temporo-parietal junction (TPJ), the medial prefrontal cortex (mPFC), the precuneus (PreCU), the temporal pole (TP) and the posterior cingulated cortex^20,21^ (PCC) in processing the social information conveyed by action kinematics^22^. Becchio and colleagues^22^ explored the role of visual kinematics in the implicit coding of intention by using functional brain imaging during the observation of grasping movements performed with social versus individual intents. The results showed stronger activation of the MNS and the MS during the observation of socially intended rather than individual movements. These findings demonstrate that, in the absence of context information, social information conveyed by action kinematics modulates intention processing: trying to read the reason why an action is performed in the absence of contextual information necessitates of a great deal of active inferencing, and the MNS may benefit from the additional recruitment of the MS^16^. Both systems seem to favor intentions arising in the context of a social interaction as opposed to individual intentions^23–29^.

With this in mind the crucial questions are whether the processing of PLD by the MNS is confined to motor simulation as previously demonstrated^14,15^, or it extends to the coding of the social involvement attached to such stimuli. And, if the latter is the case, does it work in concert with the MS? First, we hypothesize that if PLD stimuli are processed similarly to moving human like (HL), the MNS system should not distinguish between the two categories of stimuli. Alternatively, if the sparse information provided by PLD stimuli is more demanding to be coded than the coherent information provided by HL stimuli, then the MNS should be more activated for the former than for the latter stimuli. Although it has been demonstrated the involvement of the MNS in the processing of PLD stimuli, whether it is differently alerted by such stimuli in comparison to HL stimuli is still an open question. The very fact that for human displays PLD are not simply perceived as a pattern of unrelated lights is not a trivial computational problem. For instance, whereas for fixed rigid structures like a box there is only one structure that could fit the available array of moving lights, for human displays there are many rigid structures that could account for the spatial relationships between the moving lights^5^. The increase in computational demands may call for an heavier recruitment of ‘mirror’ activity.

The potential for social involvement conveyed by both PLD and HL stimuli was manipulated, by presenting PLD and HL stimuli moving towards the observer (Facing Towards, FT) or away from her/him (Facing Away, FA). We hypothesize that the potential for social involvement perceived by the observer while watching moving stimuli approaching her/him or walking away could have the ability to modulate the contribution of the MNS and the MS in the processing of PLD and HL stimuli. Specifically, we hypothesized that the activity of the MNS would be enhanced by the observation of stimuli moving towards the observer given  the preferred processing of social stimuli characterizing the MNS and its potential role in encoding potential for social involvement^23^.

To test these hypotheses, functional magnetic resonance imaging (fMRI) was used to determine the response of the MNS and the MS to different combinations of stimuli and moving directions. The patterns of response  have been explored by means of two separated analyses, focusing namely on MSN and MS brain regions. Overall, the results show that while the MS is massively recruited during the observation of HL stimuli only when the stimulus is moving towards the observer, the MNS appears to be more engaged during the observation of PLD, and to be selectively influenced by the direction of the movement.

## Results

To verify how the MS and the MNS contribute to BM processing, images of bistable PLD were presented while participants were laying in a scanner for Magnetic Resonance (MR), and were asked to judge whether the stimulus was a FT or a FA type by pressing one of two keys on a response device. HL videos were also presented, characterized by the same movement parameters as PLD. Bistability of stimuli was checked with a one-sample t-test comparing the expected (0.50 FA and 0.50 FT) and observed distribution of responses. The results confirmed the absence of any bias for giving either FA (t_(15)_ = −0.387, p = 0.704, mean = 0.491, 95% C.I. = 0.441 − 0.541), or FT (t_(15)_ = 0.387, p = 0.704, mean = 0.509, 95% C.I. = 0.459 − 0.559) responses. Comparisons between conditions were conducted on fMRI data in order to examine how the MNS and the MS responded to stimuli characterized by different biological features (PLD and HL) with a different potential for a social interaction (FT and FA). Statistical details of the results and stereotaxic coordinates of BOLD peaks are reported in Table 1.

**Table 1:** details of analysis of fMRI data.

Cluster-level		Peak-level				STRUCTURE	SYSTEM
p (FWE)	k	p (FWE)	t	MNI	SIDE		
*FT* > *FA*							
*n.s*.							
**FA** > **FT**							
<0.001	123	<0.001	5.68	−50–26 44	Left	IPL	MNS
**HL** > **PLD**							
<0.000	430	<0.001	7.45	52–54 28	Right	AG	MS
<0.001	192	<0.001	7.45	−54–50 42	Left	IPL	MNS
<0.000	256	<0.001	7.41	0 46 38	Medial	PCU	MS
<0.000	864	<0.001	7.40	− 48–56 26	Left	AG	MS
0.006	113	<0.001	6.54	−54 28 12	Left	IFG	MNS
<0.000	107	<0.001	5.36	−10 46 44	Left	mPFC	MS
<0.000	42	<0.001	5.26	14 54 40	Right	mPFC	MS
**PLD** > **HL**							
0.001	104	<0.001	7.73	4 20 44	Right	mPFC	MS
<0.001	508	<0.001	7.30	50 12 28	Right	IFG	MNS
<0.001	407	<0.001	6.84	40–40 42	Right	IPL	MNS
<0.001	177	<0.001	5.78	−32–44 48	Left	IPL	MNS
STIMULUS*DIRECTION							
0.039	84	0.011	4.53	−46 18 0	Left	IFG	MNS
0.045	82	0.029	4.14	14 40–8	Right	mPFC	MS
0.002	54	0.049	3.86	−42–42 44	Left	IPL	MNS
**POST-HOC ANALYSIS**							
*FT_PLD* > *HL*							
*n.s*.							
*FT_HL* > *PLD*							
*n.s*.							
**FA_PLD** > **HL**							
0.007	30	0.004	3.87	−40 20–2	Left	IFG	MNS
<0.000	53	<0.001	5.24	−40–40 46	Left	IPL	MNS
**FA_HL** > **PLD**							
0.041	35	<0.001	4.21	8 38–12	Right	mPFC	MS
*PLD_FT* > *FA*							
*n.s*.							
**PLD_FA** > **FT**							
0.003	76	<0.001	4.15	−44 18 2	Left	IFG	MNS
0.000	43	<0.001	5.36	−40–42 44	Left	IPL	MNS
**HL_FT** > **FA**							
0.010	32	0.005	3.79	−44 12 12	Left	IFG	MNS
*HL_FA* > *FT*							
*n.s*.							

Notes: k: cluster extent; FWE: Family Wise Error; t: t-score; MNI: Montréal Neurological Institute; FA: Facing Away; FT: Facing The viewer; PLD: Point-Light Display; HL: Human-Like; MNS: Mirror Neuron System; MS: Mentalizing System; IPL: Inferior Parietal Lobule; AG: Angular Gyrus; TPJ: Temporo-Parietal Junction; PCU: PreCUneus; mPFC: medial PreFrontal Cortex; IFG: Inferior Frontal Gyrus; NS: Not Significant.

### Main effect of movement direction (FT vs FA)

When comparing activity related to movement direction (FT vs FA) independently from the type of stimulus (PLD vs HL), no significant effects were observed within the MS system. Concerning the MNS circuit, significant differences were observed in the inferior sector of the left parietal region (IPL) for the comparison FA > FT.

### Main effect of type of stimulus (PLD vs HL)

The contrast testing for differences between PLD and HL stimuli independently from their movement direction revealed significant effects within both the MS and the MNS systems. Concerning the MS system, the contrast PLD > HL highlighted significant differential activity in the right mPFC. Although in a more dorsomedial region, also the opposite contrast (HL > PLD) was associated with significant effects in the mPFC bilaterally, together with the bilateral AG and the right PCU. For the MNS circuit the comparison PLD > HL highlighted significant differences in the IPL bilaterally, and within the right IFG. The opposite contrast, HL > PLD, highlighted significant differences in the left hemisphere, within the IPL and the IFG.

### Interaction between movement direction and type of stimulus

**Figure 1:**
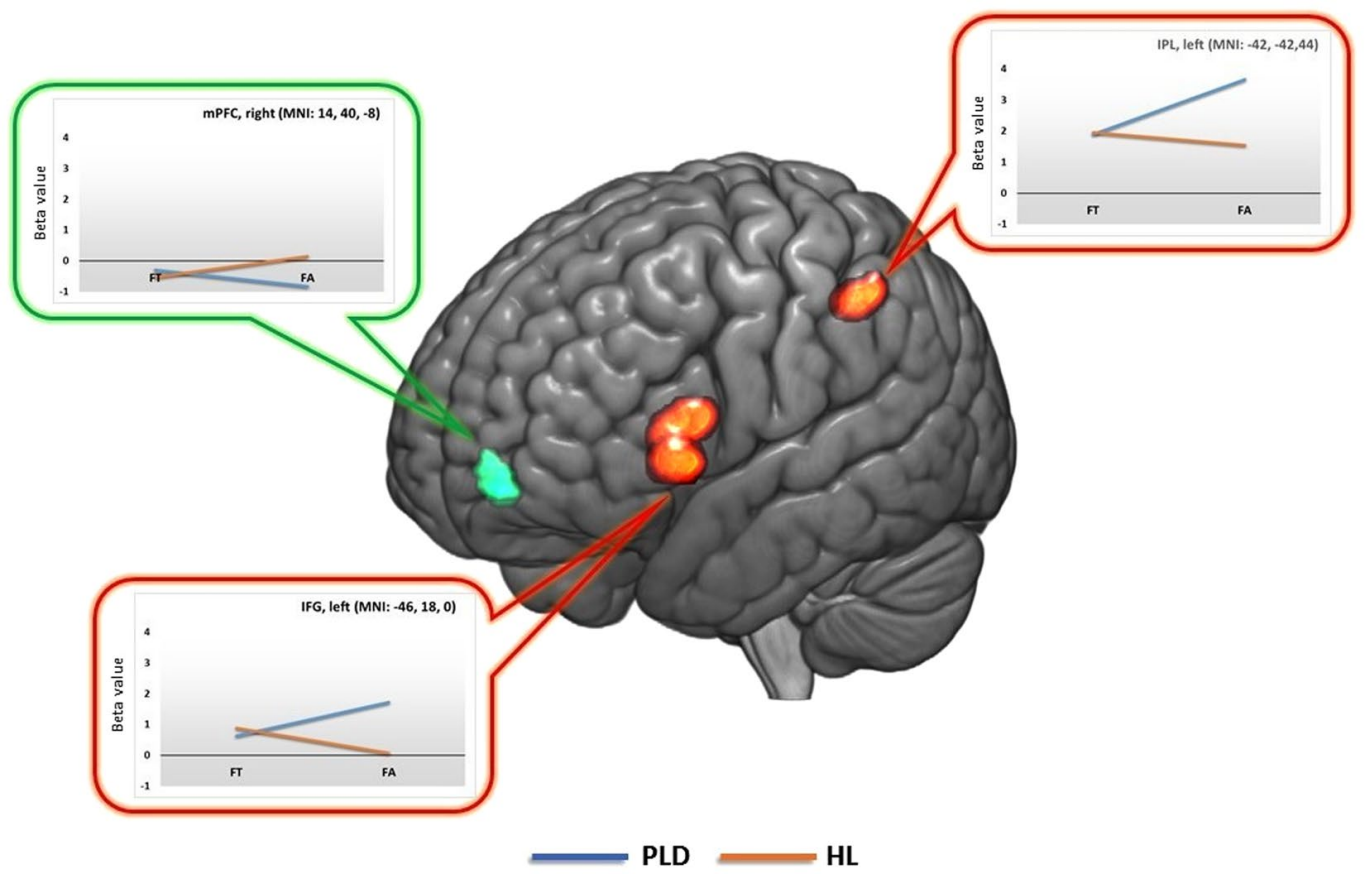
interaction effects between the type of stimulus and movement direction. mPFC: medial PreFrontal Cortex; IFG: Inferior frontal Gyrus; IPL: Inferior Parietal Lobule; MNI: Montréal Neurological Institute; PLD: Point-Light Display; HL: Human-Like; FT: Facing Towards; FA: Facing Away. Images are presented in neurological convention using the template provided by the software Mricron_GL (http://www.cabiatl.com/mricrogl/).

The interaction between the type of stimulus (PLD vs HL) and its movement direction (FT vs FA) revealed significant differential activity within both the MS and the MNS. Concerning the MS the right mPFC appeared to be sensitive to the different combinations of movement direction and type of stimulus, as well as the left IFG and IPL belonging to the MNS. Significant effects were explored by conducting post-hoc contrasts (paired *t*-tests) testing for differences between single conditions within the aforementioned brain locations (see Table 1). This procedure highlighted that the right mPFC was significantly more involved by HL rather than PLD stimuli, but only in the FA condition (see Table 1 and Fig. 1). When considering the MNS, both the left IFG and the left IPL were significantly more involved while watching PLD rather than HL, but only when the stimuli were moving away from the observer (FA). In addition, both regions appeared to be more engaged during the observation of FA with respect to FT stimuli, but only for PLD stimuli. No further significant differences emerged within the considered regions.

## Discussion

The experiment consisted of short videoclips representing either bistable PLD or videos of human walkers (HL) moving towards or away from the observer. While observing such stimuli, participants were requested to judge whether they were moving towards or away from them by pressing a key on a response device. Results showed a significant modulation of BOLD signal within the network of areas belonging to both the MNS and the MS, revealing different patterns of response according to the different combinations of type of stimulus and moving direction, suggesting that MNS and MS systems are differentially activated by PLD and HL stimuli. Furthermore, only the MNS appears to be modulated by stimulus direction, which directly flows into a key question at the roots of the present investigation, that is whether the MNS is concerned with the processing of social cues conveyed by PLD stimuli.

Saygin and colleagues^14^ suggest that the observation of point-light biological motion recruits significantly stronger activity in the frontal region in respect to scrambled movement. Along the same line, Ulloa and Pineda^15^ revealed that point-light biological motion engages the MNS as reflected in *mu* rhythm suppression observed in response to biological but not scrambled motion. Overall, it seems that the motor system of the observer may be recruited to “fill in” these simplified displays, in a manner similar to the way mirror neurons are activated in order to assist in action understanding. Our results not only confirm that the MNS is recruited during the observation of PLD, but also that it is sensitive to the moving direction of the observed stimulus. Its contribution becomes evident when considering a stimulus moving away from the observer (FA condition), but not when the stimulus faces the observer (FT condition). These findings support the hypothesis that the mirror mechanism could play a crucial role in mediating the process of integrating sparse motion cues into a complete human action. In other words, brain regions, whose role in social interaction has been widely explored and testified^13–15^, seem to play a major role when the potential for a social involvement has to be inferred. As outlined in previous work^28–31^, we are more used to interact with other individuals in a face-to-face rather than a face-to-back situation. In the latter case ascribing to a stimulus the potential for social involvement might be more demanding and might require additional ‘efforts’ by the MNS to ‘socially’ interpret ambiguous social cues (as those provided by PLD in the FA condition) rather than coding more evident social cues (as those provided by HL stimuli in the FT condition). In this perspective, the MNS seems to carry the additional processing weight for stimulus interpretation, confirming its role in action understanding^32^. This finding could be explained also by a strong tendency to interpret stimuli representing a walker moving as facing the viewer even when they are not^27^. Several factors could be responsible for this perceptual bias. For example, high-level cognitive factors, as suggested by Klopfer^28^, or stored motion patterns (‘sprites’^31^). In these accounts familiarity is a key factor, and it is assumed that observers are confronted visually more often with frontal than with back views of their conspecifics. The biological and/or the potential for social interaction linked to frontal views might also be at the heart of the phenomenon. Even if the present study does not allow us to make definite claims as to the source of this tendency, the literature indicates that observers tend to interpret an upright walker presented in different views as approaching^27^.

The key role of MNS in coding for PLD stimuli becomes evident also when comparing PLD to HL in FA condition: the representation of an entity walking away from the observer provided by moving point lights involves the MNS more than a HL moving in the same direction. This finding adds to a rather slim literature looking at the possible role of the MNS as a filtering mechanism for social kind of information and suggests that activity of the MNS is dependent upon whether the agent is facing towards or away from the observer^33^.

The contribution of the MNS is not only confined to the processing of PLD stimuli, but it emerges also for HL stimuli when considering movement direction. The MNS is significantly more recruited in the FT rather than in the FA condition. At first sight, this result may contrast with the finding that, for PLD stimuli, activity within the MNS is greater for the FA than for the FT condition. However, the fact that the contribution of the MNS becomes significant when the HL stimulus approaches the observer, presumably reflects the coding of social aspects provided by a context in which a clear cue indicating the potential for social interaction is presented^13–15^. In other words, the idea that activations within the MNS are enhanced when watching a scene with a potential for social interactions appears to be confirmed^23^.

The contribution of the MS appears to be significant when comparing HL and PLD stimuli moving away from the observer. This pattern of results supports the idea that mentalizing regions contribute to analyze other people’s actions when the viewer decides to reflect upon their goals and intentions^34,35^. It seems that, although PLD convey surprisingly detailed information about movements of the human body, they might be too simplified to require a concrete involvement by the MS. In this perspective, the recruitment of MS regions during the observation of moving biological stimuli (TPJ, mPFC, PCU) is coherent with the results obtained by a variety of mentalizing tasks such as the attribution of goals, intentions and the monitoring other-executed actions^22,36–39^. We also know from our everyday experience that when someone has his/her back turned the potential for a social interaction in such situation is much reduced compared with a situation of facing someone. Therefore, we suggest that the pattern of activity within the MS in the forward facing might reflect a process modulated by the social intention ascribed to the person being observed. Because reflecting on the intention of an agent performing an action necessarily involves the processing of what s/he is doing and how s/he is doing it, activation within MS varies as a function of intention-related information conveyed by the observed action^22^.

## Conclusion

The perception of point-light stimuli remains an impressive achievement of our visual system. The ambiguous PLD might provide a valuable new window on the issue whether the perception of human action is ‘special’, in the sense that actions are processed in a qualitatively unique way and whether this functional specialization is supported by a specific architecture hard-wired in the brain. The present results demonstrate that the recruitment of the MNS is crucial in coding PLD but only when considering stimuli moving away from the observer. This confirms the crucial role of the MNS in the processing of abstract visual representations of actions defined by motion cues alone. Observing HL, instead, seems to elicit an involvement of the MS, with a less pronounced contribution of the MNS suggesting a specific role of the MS in coding for the social meaning conveyed by human-like moving stimuli. In this view the mirror and the mentalizing systems are portrayed as sensitive to different aspects of social involvement, playing specific roles depending on the nature of the observed stimulus.

## Methods

### Participants

Sixteen participants took part in the experiment (10 female, mean age 22,75 years, SD 2,9). They all had normal or corrected-to-normal vision, no neurologic or psychiatric history, and were not under medical treatment with drugs potentially interfering with cerebral blood flow and/or brain metabolism. Before entering the scanner all participants underwent MR safety screening and gave their written consent to the MR scanning procedure. Information concerning experiment aims and confidentiality criteria for personal and MR data treatment was also provided, and participants were required to give their consent to take part in their study and to allow the experimenter to use their data for scientific dissemination (e.g., scientific articles). The study was approved by the Ethics Committee for Clinical Experimentation of the University of Padova (CESC - Comitato Etico per la Sperimentazione Clinica, University of Padova). All experimental procedures were carried out in accordance with relevant guidelines and regulations.

### Stimuli and task

**Figure 2:**
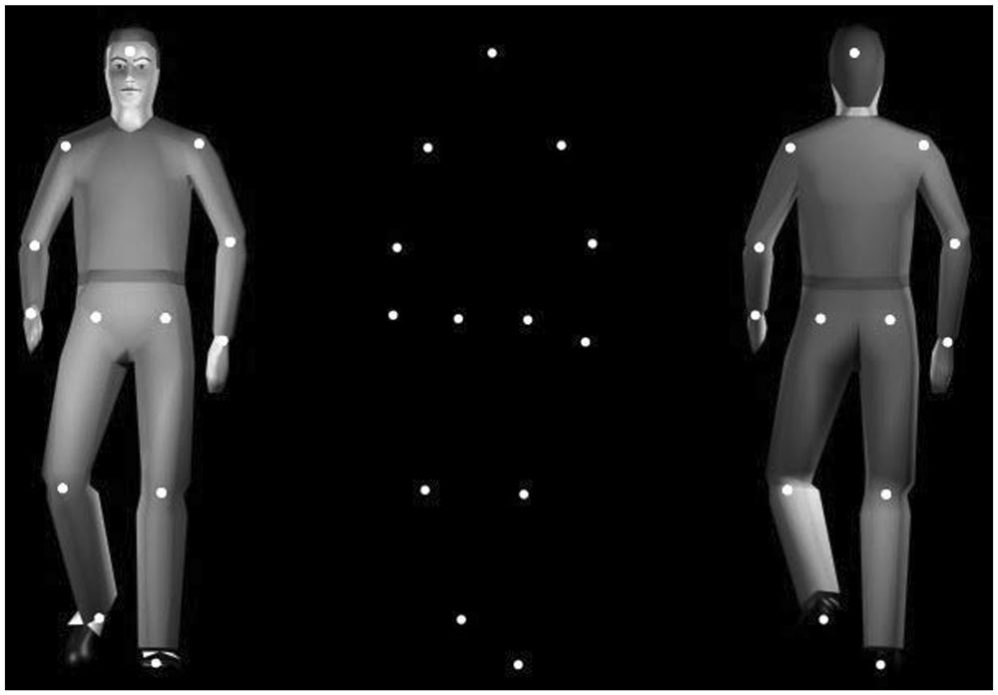
The central panel depicts bistable point-light displays (PLD) and the two possible ways to interpret its motion direction: Facing The viewer (FT) on the left, and Facing Away (FA) on the right.

Stimuli consisted of PLD of a walking person with 13 markers indicating the head and the center of the major joints of a person (shoulders, elbows, wrists, hips, knees, and feet; see Fig. 2). All PLD were derived from the stimulus set of Vanrie and Verfaillie^40^, consisting of 3D-coordinates of a human actions walker of which the translational component was removed^41^. As a result, the walker moved as if walking on a treadmill, in the center of the screen. The visual angle between the points attached to the head and the feet was about 5.7 deg. Each dot subtended about 0.14 deg. Each stimulus presentation lasted 2.885 s consisting of two walking cycles, each consisting of two steps. The starting posture in the walking cycle was randomized across trials. Stimuli were black against a grey background, and were administered using Presentation software (14.4 version, Neurobehavioral Systems) on a 15.4-inch monitor (VisuaStim XGA, Resonance Technology Inc., display resolution: 1280 × 800; refresh rate: 60 Hz). In order to obtain stimuli with different degrees of ambiguity in respect to their orientation (FT vs. FA), the in-depth orientation of the point-light figures was manipulated by introducing perspective cues, including modifications of shape, size, and position of the dots as a result of a perspective projection^40^. Levels of perspective manipulation (corresponding to 30%, 50%, and 70% FT responses) were determined individually for each participant during a preliminary adjustment task (see below). This means that, for each participant, we employed PLD *subjectively* ambiguous in respect to their orientation. HL stimuli were characterized by the same movement parameters as the PLD (see Fig. 2). For HL stimuli, six unique video-clips (three FT and three FA videos) were recorded form three ‘actors’ with minor physical differences (e.g., haircut or wearing details). Actors were positioned at the center of a corridor and then asked to walk either towards the video camera positioned in front of them (FT), or in the opposite direction (FA). Video-clips were then edited using Adobe Premiere Pro CS5 (.wmv format, disabled audio, 25 frames/s) in order to produce stimuli in which the actors moved as if walking on a treadmill, in the center of a gray background. Once the video-clip was edited, a ‘human-like’ digital actor was clearly recognizable but without allowing for the identification of the ‘original’ actor.

### Preliminary Adjustment Task

Despite the absence of differentiating visual cues, when presented with an orthographic projection of a PLD, observers tend to show a tendency to interpret the stimulus as facing towards them^40–42^. To obtain subjectively ad-hoc bistable stimuli, before scanning the point of subjective ambiguity^42^ (PSA) was determined for each participant. PSA falls when a stimulus with a specific degree of perspective manipulation is interpreted by a specific individual 50% of times as FT and 50% of times as FA. According to Schouten and Verfaillie^42^, the perspective cues carrying the information concerning the veridical orientation of the point-light walker were gradually manipulated across different levels (nine), from moderately FT to strongly FA. The nine levels of perspective information included one orthographic projection (neutral) and eight distance manipulations of the convergence point, corresponding to −4, −8, −16, 16, 8, 6, 4, and 2 times the height of the walker (negative values indicate FT stimuli). This resulted respectively in field of view angles of −53°, −28°, −14°, 14°, 28°, 37°, 53°, and 90°.

On each trial, participants were asked to indicate whether the visually presented stimulus was perceived as oriented towards or away from them by pressing one of two buttons of a response box (the button below the index finger for FT and the button below the middle finger for FA responses). Participants were instructed to respond according to their own subjective experience and it was stressed that an equal distribution of both response alternatives was not necessary. In total, each participant completed 270 trials (9 perspective levels * 30 repetitions). Individual perspective levels were determined by fitting a cumulative Gaussian function to the proportion of FT responses and selecting the 30%, 50% (PSA), and 70% thresholds. The lowest and highest perspective manipulations allowed were −1 (−127 degrees) and 1 (127 degrees).

### Experimental design

The whole experimental session consisted in 6 functional runs where the type of moving stimulus (PLD vs HL) was blocked across runs, with a total of 3 runs for each condition (6 runs in all). Runs belonging to the same type of stimulus were presented consecutively (PLD, PLD, PLD, HL, HL, HL or HL, HL, HL, PLD, PLD, PLD), counterbalancing the sequence across participants. 135 trials per condition were administered with an event related design, for a total of 270 trials. Concerning PLD stimuli the trials were equally divided into FA, and FT (45 trials per each individual perspective threshold). The 135 HL stimuli were also divided into 67 or 68 FA and 67 or 68 FT trials depending on the participant… Each trial started with the presentation of the moving stimulus (2885 ms). Then participants laying in the MR scanner were instructed to decide whether the visually presented stimulus was oriented towards or away from them. A screen informed the participant about the key associated with the response. The Italian word ‘di fronte’ (FT) on the left prompted a button press of a MR compatible response box with the index finger, while the word ‘di spalle’ (FA) on the right prompted a button press with the middle finger. Participants were asked to respond as quickly as possible while keeping the number of errors low. This screen was presented for at most 3000 ms, or until response. It was then followed by an interstimulus interval (black fixation cross) with a duration varying from 1 to 6 seconds, distributed according to a ‘long exponential’ probability distribution^43^ and randomized across trials.

### fMRI data acquisition

Data were acquired with a 1.5 T Siemens Avanto MR scanner (Siemens Medical Systems, Erlangen, Germany) equipped with a standard eight channels coils. Participants were positioned headfirst and supine in the magnet bore. The head was held in place with clamps to avoid head motion. Functional images were acquired with a gradient-echo, Echo-Planar (EPI) T2*-weighted sequence in order to measure blood oxygenation level-dependent signal (BOLD) throughout the whole brain (37 contiguous axial slices, ascending interleaved sequence, 56 × 64 voxels, 3.5 mm × 3.5 mm × 4.0 mm resolution, FOV = 196 mm × 224 mm, flip angle = 90°, TE = 49 ms). Volumes were acquired continuously for each run with a repetition time (TR) of 3 s; 134 volumes were collected in each single scanning run, resulting in 6 functional runs of 6 min and 42 s each. High-resolution anatomical images were acquired for each participant using a T1-weighted 3D MPRAGE sequence (176 axial slices, no interslice gap, data matrix 256 × 256, 1 mm isotropic voxels, TR = 1900 ms, TE = 2.91 ms, flip angle = 15°).

### fMRI data preprocessing

Data preprocessing was performed by combining Statistical Parametric Mapping 12 (SPM - http://www.fil.ion.ucl.ac.uk/spm), FMRIB Software Library (FSL - https://fsl.fmrib.ox.ac.uk/fsl) and Advanced Normalization Tools (ANTs - http://stnava.github.io/ANTs). Functional images were motion corrected with FSL, by aligning each volume to the median one in each session using an affine transformation. The six median volumes were also used to create a template by using the buildtemplateparallel script in ANTs^44,45^, which also returned the linear transformations from each single median volume to the template. A linear transformation was calculated from the template to the T1 image using the Boundary-Based Registration^46^ (BBR) implemented in FSL. The motion corrected EPI images were normalized to the MNI152 space in a single step by concatenating the three transformations (EPI ->EPI-template, EPI-template ->T1, T1 - >MNI152). The normalized EPI images were then spatially smoothed with SPM12 with a Gaussian kernel of 8 mm^3^ FWHM (number of resels: 38 for MNS, 35 for MS), and temporally filtered with a high-pass filter with a frequency cut-off of 1/128 Hz. Finally, FSL was adopted to detect the timepoints in the fMRI series that were corrupted by large motion. Structural T1 images were initially corrected for bias field inhomogeneities using the N4 algorithm^47^ and subsequently segmented in Cerebro Spinal Fluid (CSF), WM and GM images. Then a transformation from T1 to MNI152 (1 mm^3^ isovoxel) was calculated using a non-linear diffeomorphic deformation. All these steps were performed using ANTs^44,45,47^.

### fMRI data analyses

**Figure 3:**
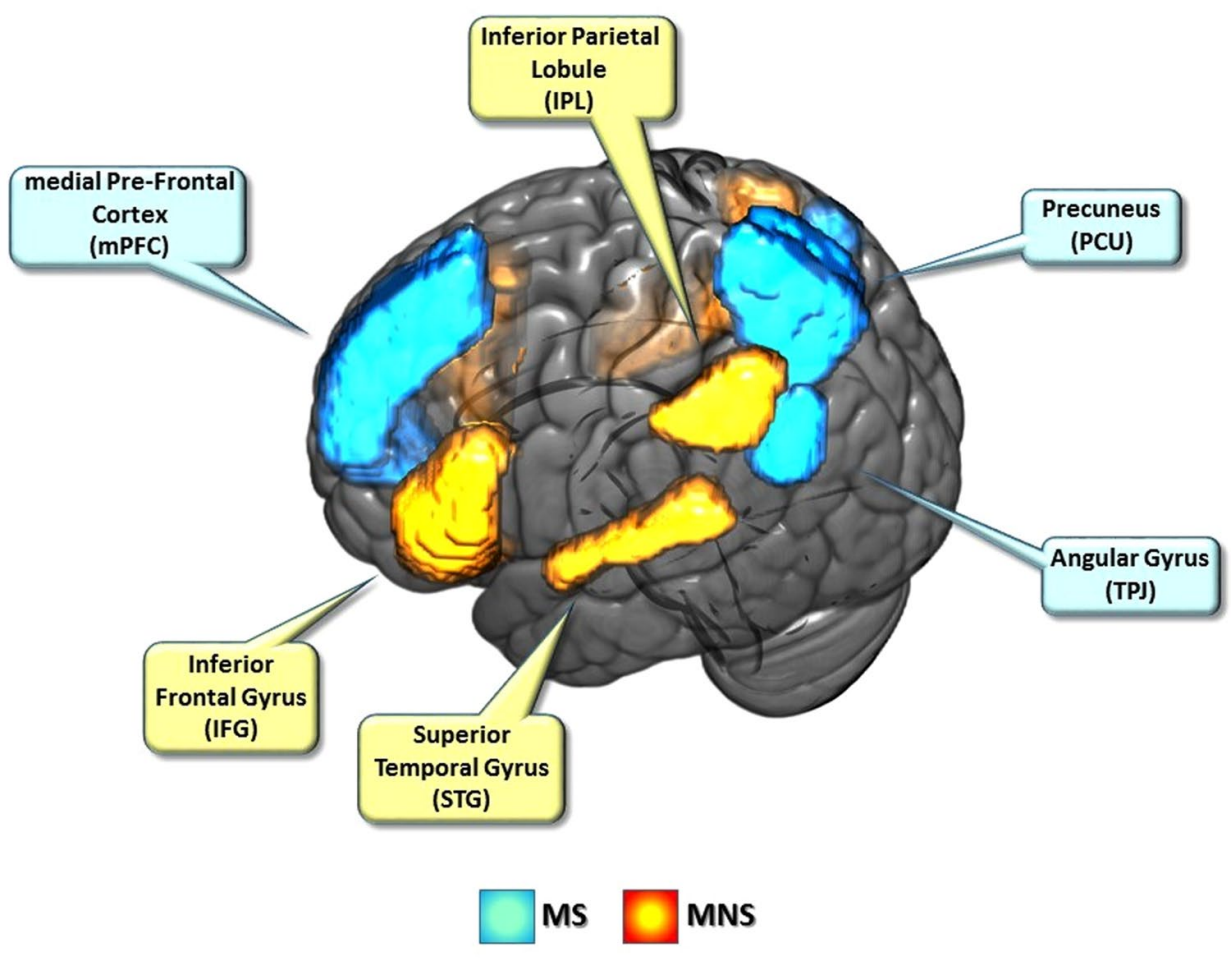
Brain regions considered for the Mirror Neuron Systems (MNS) and the Mentalizing System (MS) masks (both hemispheres).

fMRI data analysis of time series was performed with SPM12. BOLD signal was first analyzed at the individual level: for each participant, separate regressors were defined based on the timing of presentation of each of the four experimental conditions (PLD_FT, PLD_FA, HL_FT, HL_FA), and these functions were convolved with a canonical, synthetic hemodynamic response function (HRF). Each trial was modelled as a 2.885 s duration event, starting at the onset of the video. The time window devoted to question presentation regarding movement direction (3000 ms or until response) was modeled as regressor of no interest, as well as head motion parameters (6) calculated during the realignment stage, and eventual additional matrices controlling for nuisance introduced by corrupted volumes. Finally, stimuli classified as FT or FA on the basis of the individual preliminary adjustment task but receiving a different response during the proper experiment were labeled as ‘errors’ and modeled as additional regressors. For each participant, all regressors were incorporated into a General Linear Model^48^ (GLM). Individual models were separately estimated and contrasts were defined in order to pick out the main effects of each experimental condition. At the second level, contrasts for the main effect of type of movement direction (FT or FA) and the video type (PLD or HL) were obtained for the whole brain. Since the study aimed at describing the response of the MNS and the MS to our experimental manipulation, two ANOVAs were conducted, considering different sets of brain regions. Two different masks were built: for the MNS the Inferior Frontal Gyrus (IFG, pars opercularis and triangularis), the Inferior Parietal Cortex (IPC) and the Superior Temporal Gyrus (STG) were considered (see Fig. 3). For the MS circuit the Precuneus (PCU), the medial PreFrontal Cortex (mPFC) and the Temporo-Parietal Junction (TPJ) were included (see Fig. 3). Concerning TPJ, its more posterior sector was considered, corresponding to the Angular Gyrus (AG): recent work has underlined that activations related to social cognition mainly fall in this region^49^. Functional resting state connectivity between the AG and other MS regions such as mPFC and PCC has also been described^50^. The Wake Forest University brain atlas (http://fmri.wfubmc.edu/software/pickatlas) was used to create both MNS and MS masks. These two masks were adopted as searching areas in the analysis of variance and the subsequent post-hoc analysis, considering movement direction (FT vs FA) and type of stimulus (PLD vs HL) as within-subject variables. Results obtained through this masking procedure were thresholded at p < 0.05 (FWE corrected for multiple comparisons) at both the cluster and peak levels (cluster extent of ≥30 voxels).
